# Geographical distribution of *Anopheles stephensi* in eastern Ethiopia

**DOI:** 10.1186/s13071-020-3904-y

**Published:** 2020-01-20

**Authors:** Meshesha Balkew, Peter Mumba, Dereje Dengela, Gedeon Yohannes, Dejene Getachew, Solomon Yared, Sheleme Chibsa, Matthew Murphy, Kristen George, Karen Lopez, Daniel Janies, Sae Hee Choi, Joseph Spear, Seth R. Irish, Tamar E. Carter

**Affiliations:** 1 Abt Associates, PMI VectorLink Ethiopia Project, Addis Ababa, Ethiopia; 2 Abt Associates, PMI VectorLink Project, Rockville, MD USA; 3 Dire Dawa University, Dire Dawa, Ethiopia; 4 Jigjiga University, Jigjiga, Ethiopia; 5 US President’s Malaria Initiative (PMI), Addis Ababa, Ethiopia; 6 United States Agency for International Development (USAID), Addis Ababa, Ethiopia; 7 Division of Parasitic Diseases and Malaria, Center for Global Health, Centers for Disease Control and Prevention, Atlanta, GA USA; 8 Bureau for Global Health, Office of Infectious Disease, Malaria Division, USAID, Arlington, VA USA; 9 University of North Carolina at Charlotte, Charlotte, NC USA; 10 Baylor University, Waco, TX USA

**Keywords:** *Anopheles stephensi*, Ethiopia, Malaria, Horn of Africa

## Abstract

**Background:**

The recent detection of the South Asian malaria vector *Anopheles stephensi* in Ethiopia and other regions in the Horn of Africa has raised concerns about its potential impact on malaria transmission. We report here the findings of a survey for this species in eastern Ethiopia using both morphological and molecular methods for species identification.

**Methods:**

Adult and larval/pupal collections were conducted at ten sites in eastern Ethiopia and *Anopheles* specimens were identified using standard morphological keys and genetic analysis.

**Results:**

In total, 2231 morphologically identified *An. stephensi* were collected. A molecular approach incorporating both PCR endpoint assay and sequencing of portions of the internal transcribed spacer 2 (ITS2) and cytochrome *c* oxidase subunit 1 (*cox*1) loci confirmed the identity of the *An. stephensi* in most cases (119/124 of the morphologically identified *An. stephensi* confirmed molecularly). Additionally, we observed *Aedes aegypti* larvae and pupae at many of the *An. stephensi* larval habitats.

**Conclusions:**

Our findings show that *An. stephensi* is widely distributed in eastern Ethiopia and highlight the need for further surveillance in the southern, western and northern parts of the country and throughout the Horn of Africa.

## Background

Malaria remains a leading global health concern with over 250 million cases reported yearly [1]. In Ethiopia, even though there has been steady progress in the reduction of malaria [2], 1.5 million cases were reported in 2018 [1]. Developing effective malaria control strategies in Ethiopia require knowledge of local mosquito vector species [3]. One threat to continued progress against malaria is the expansion of vectors into new areas. The South Asian vector *Anopheles stephensi* was recently discovered in Ethiopia and is raising concerns about the impact on malaria transmission in the country and the rest of the Horn of Africa. *Anopheles stephensi* is a major malaria vector in South Asia and the Middle East, including the Arabian Peninsula [4], and is known to transmit both the major malaria parasite species *Plasmodium falciparum* and *P. vivax* [5, 6]. The first report of *An. stephensi* in the Horn of Africa was from Djibouti in 2013 [7] and was recently confirmed to be persisting in the country [8]. *Anopheles stephensi* was detected in Ethiopia for the first time in 2016 in Kebridehar (Somali Region) but it remains unclear how broadly distributed the species is in the rest of the country [9].

Understanding the distribution of *An. stephensi* in Ethiopia is critical to evaluating the threat it poses to malaria control in Ethiopia and the rest of the Horn of Africa [9]. It is important during initial surveillance of a potential new vector to evaluate the accuracy of species identifications. Genetic analysis can be a useful complement to morphological identification to achieve optimal accuracy in species identification [10], particularly when identifying a recently detected species. The objective of the study was to investigate the geographic distribution of *An. stephensi* in north eastern and eastern urban localities in Ethiopia using morphological and molecular identification of wild-caught *Anopheles*.

## Methods

### Survey sites

**Fig. 1:**
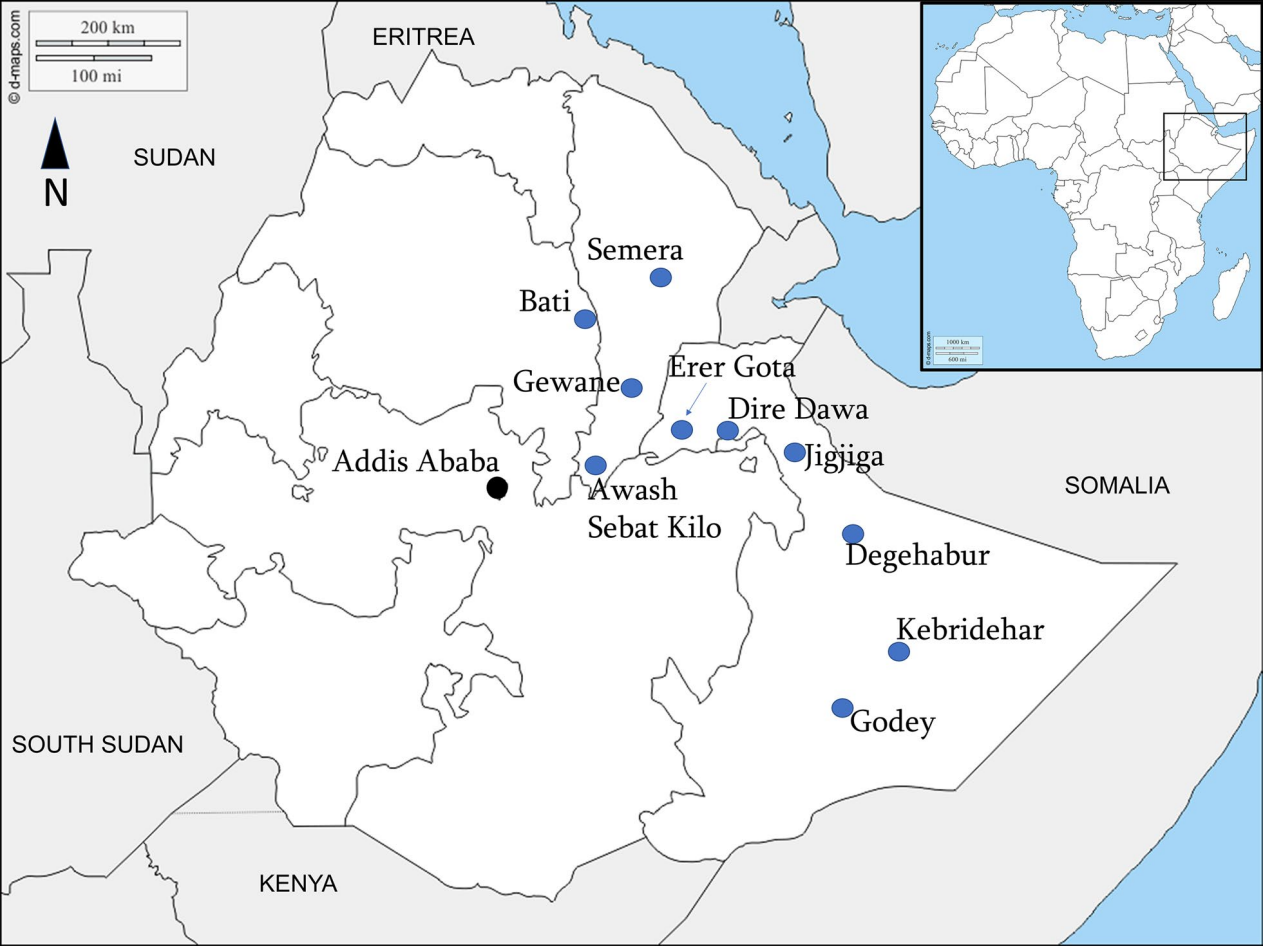
Map of study sites in Ethiopia

*Anopheles stephensi* surveys were conducted from August to November 2018 in ten selected urban sites situated in a climatic zone of either tropical, hot semi-arid or desert with an elevation range of 294 to 2055 meters above sea level. The localities included five in Somali region, three in Afar, one in Amhara region, and Dire Dawa city (Table 1, Fig. 1).

**Table 1:** Collection site altitude and geographical coordinates

Site	Region	Altitude (masl)	Coordinates
Jigjiga	Somali	1657	9°3′51″N, 42°7′93″E
Erer Gota	Somali	1090	9°5′56″N, 41°3′84″E
Kebridehar	Somali	532	6°7′38″N, 44°2′77″E
Godey	Somali	294	5°9′49″N, 43°5’53″E
Degehabur	Somali	1065	8°2′23″N, 43°5′58″E
Semera	Afar	431	11°7′94″N, 41°0′08″E
Gewane	Afar	617	10°1′66″N, 40°6′46″E
Awash Sebat Kilo	Afar	916	8.9′89″N, 40°1′64″E
Dire Dawa	Dire Dawa	1178	9°5′96″N, 41°8′54″E
Bati	Amhara	2055	11°1′92″N, 40°0′17″E

*Abbreviation*: masl, meters above sea level

The areas have mean annual temperatures of about 20 °C to 30 °C and a mean annual rainfall from 200 to 900 mm. There is a smaller rainy season between March and May, followed by a longer period between July and October [11].

### Sampling of *An. stephensi*

For the purpose of identification of *An. stephensi* and consequently to determine its presence in the study sites, free flying adult sampling was done together with raising of adults from larval and pupal collections.

#### Adult sampling

The entomological methods to sample adult mosquitoes were pyrethrum spray sheet collections (PSC) and Centers for Disease Control (CDC) light traps. In each site, PSC was conducted in 30 houses and CDC light traps were set in 20 houses for one night per house. Prior to PSC operation, consent was obtained from heads of households and PSC activities were conducted from 6:30 h to 8:00 h mostly in sleeping rooms of big houses and additionally in living rooms of small houses. Houses for PSC were prepared following the WHO standard guidelines. All food and drink was removed, children and small animals made to stay outdoors, and non-removable items together with the floor were covered with pieces of white cloth sheet. All eaves, openings in windows and other mosquito exit points were blocked with pieces of cloth, as much as possible. Two operators, one inside and the other outside of the room/house were assigned to spray an aerosol insecticide (Baygon, SC Johnson & Son Inc, Racine, Wisconsin, USA). Wearing a protective nose mask, the operator who was outside the house sprayed the aerosol walking around the house to drive in escapee mosquitoes while the other operator sprayed the insecticide in the whole room by moving from left to right of the door. Then the room/house was closed for ten minutes and knocked down mosquitoes were collected from the ground cloth using forceps and placed in Petri dishes.

CDC light trap collections were made from 18:00 h to 6:00 h. A CDC light trap was hung 1.5 m above the floor and close to a sleeping location where the occupants are protected with LLINs. Trapped mosquitoes were transferred from collection bag to cages. Those alive were killed with chloroform.

PSC and CDC light trap sampled mosquitoes were sorted into culicines and *Anopheles*. The revised morphological key of Gillies & Coetzee [12] together with a key prepared by Maureen Coetzee (unpublished) were used to discriminate *An. stephensi* from other *Anopheles* spp. and *An. arabiensis* in particular. The morphological features used were: speckling on palps, base and apex of palp four with white scales; absence of upper proepisternal setae on the thorax and 3^rd^ main dark area (preapical dark spot) of wing vein R1 with no pale interruption; and having two pale spots on the second main dark area of the costa. Mosquitoes identified as *An. stephensi* were stored individually in Eppendorf tubes with silica gel to ensure the mosquitoes were kept dry for subsequent molecular analysis.

#### Larval and pupal sampling

Larvae and pupae of *Anopheles* were dipped from likely larval breeding habitats including man-made water containers, freshwater pools, stream margins, discarded tires, plastic containers and irrigation ditches. Water storage for household use and construction is common in the sites. These include metal and plastic tanks, cisterns, barrels and plastic sheets hung from polls. In the Somali region, water is stored in a container locally called “Birka” and it is constructed from cement and stone.

Larvae were reared in field insectaries using water taken from breeding habitats, feeding them with baking yeast and exposing them to sunlight during the day. Pupae were transferred into adult emergence cages and adults were identified to species using the keys described above; the specimens of *An. stephensi* were preserved as described above.

### Molecular identification of species

To confirm morphological species identifications, a subset of the *An. stephensi* specimens were molecularly characterized. Additional *An. gambiae* (*s.l*.) specimens were also analyzed as controls for comparison. Species identification was completed using two approaches: (i) PCR endpoint assay utilizing the internal transcribed spacer 2 (ITS2) locus; and (ii) sequencing portions of ITS2 and cytochrome *c* oxidase subunit 1 (*cox*1). ITS2 endpoint assay was performed as previously described [13] using the primers 5.8SB (5′-ATG CTT AAA TTT AGG GGG TAG TC-3′) and 28SC (5′-GTC TCG CGA CTG CAA CTG-3′) and the following modifications: final reagent concentrations and components were 0.5 μM for each primer; 1× Promega GoTAQ HotStart master mix (Promega, Madison, Wisconsin, USA); and water for a total reaction volume of 25 µl. The temperature protocol was performed as follows: 95 ℃ for 1 min, 30 cycles of 96 ℃ for 30 s, 48 °C for 30 s, 72 ℃ for 1 min, and a final extension step at 72 ℃ for 10 min. *Anopheles stephensi* specimens were identified by visualization of 522-bp band with gel electrophoresis; non-*An. stephensi* specimens do not amplify and no band is present. Portions of the ITS2 and *cox*1 loci were also amplified for sequencing using previously detailed methods [14]. PCR products were sequenced using Sanger technology with ABI BigDyeTM terminator v3.1 chemistry (Thermofisher Scientific, Santa Clara, CA, USA) according to the manufacturerʼs recommendations and run on a 3130 Genetic Analyzer (Thermo Fisher Scientific). Sequences were cleaned and analyzed using CodonCode (CodonCode Corporation, Centerville, MA, USA). ITS2 and *cox*1 sequences from *Anopheles* specimens were submitted as queries to the National Center for Biotechnology Information’s (NCBI) Basic Local Alignment Search Tool (BLAST) [15] against the nucleotide collection in NCBI’s GenBank under default parameters (max high-scoring segment pairs (HSP) 250, expect threshold 10, word size 28, optimized for highly similar hits, not specific to any organism). The *Anopheles* subject sequences from NCBI that formed HSP with the queries were used as the basis of species identification. The percent of species correctly identified using morphology was calculated using the molecular data for comparison.

### *Plasmodium* detection

Wild-caught adult *An. stephensi* were screened for *P. falciparum* and *P. vivax* DNA as an indication of *Plasmodium* infection. PCR amplification targeted the *SSU* RNA gene with species-specific primers using a previously published approach [16]. Presence of a band was a positive indication of *Plasmodium* DNA in the sample. *Plasmodium falciparum* and *P. vivax SSU* RNA positive controls were included for each reaction set (Microbiologics, St. Cloud, MN, USA).

## Results

A total of 82 adult *An. stephensi* from 300 PSCs and 200 CDC light traps were collected in 7 of the 10 sites. The sites with no adult collections were Jigjiga, Awash Sebat Kilo and Bati (Table 1). Of the 82 adults 81.7% (*n* = 67) were from PSC and the remaining 18.3% (*n* = 15) were from CDC light traps. The majority of *An. stephensi* sampled using PSC were from Semera and Erer and that of CDC were from Degehabur. Larval and pupal collections yielded 2149 adult *An. stephensi* from all the sites confirming the presence of immature stages (Table 2).

**Table 2:** Number of adult *An. stephensi* collections from PSC, CDC traps, and larval and pupal collections

Region	Site	Method of collections and *An. stephensi*			
		PSC	CDC	Adults raised from larvae and pupae	Total
		*n* (%)	*n* (%)	*n* (%)	
Somali	Jigjiga	0 (0)	0 (0)	18 (100)	18
	Erer Gota	14 (9.8)	0 (0)	129 (90.2)	143
	Kebridehar	3 (0.4)	0 (0)	700 (99.6)	703
	Godey	2 (0.6)	0 (0)	340 (99.4)	342
	Degehabur	1 (0.6)	13 (7.2)	166 (92.2)	180
Afar	Semera	38 (12.7)	1 (0.3)	260 (87.0)	299
	Gewane	6 (5.5)	1 (0.9)	102 (93.6)	109
	Awash Sebat Kilo	0 (0)	0 (0)	26 (100)	26
Dire Dawa	Dire Dawa	3 (1.1)	0 (0)	277 (98.9)	280
Amhara	Bati	0 (0)	0 (0)	135 (100)	135
Total		67	15	2149	2231

*Abbreviation*: n, number collected

**Fig. 2:**
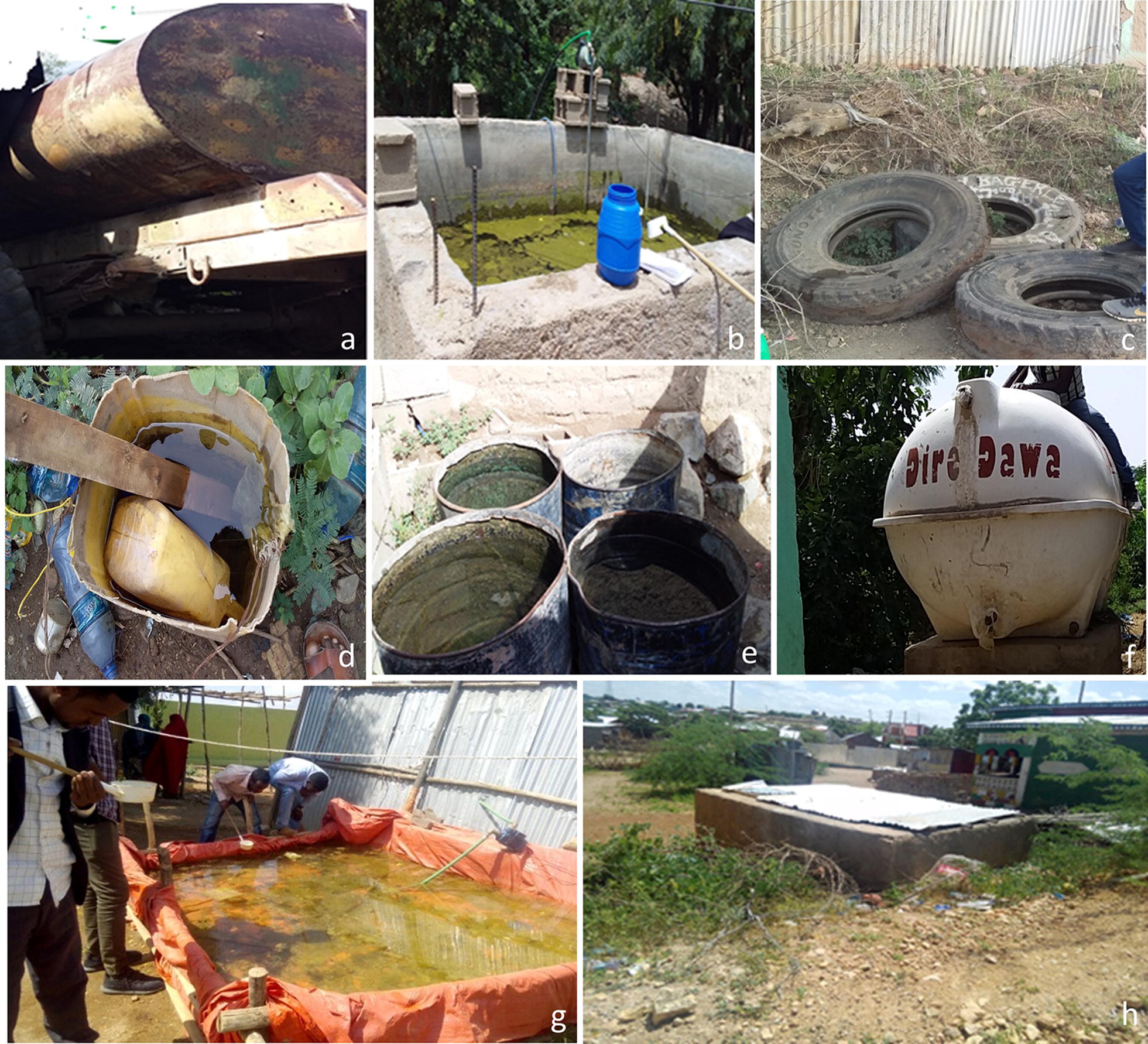
Examples of breeding sites where *An. stephensi* larvae and pupae were found: **a** water tank trucks; **b** construction water storage reservoirs; **c** discarded tires; **d** buckets; **e** steel drums; **f** water tanks; **g** temporary water storage reservoirs; **h** birkas

Larval breeding habitats included concrete water cisterns, discarded tires, water tanks, steel drums, plastic sheet water storage at construction sites, discarded buckets, abandoned car wash and discarded vehicle part (Fig. 2). The potential breeding containers in each of the sites are presented in Table 3. However, since this was a onetime cross-sectional survey, the likelihood of missing other breeding sites is inevitable. Moreover, the containers found without larvae at the time of the survey might be positive another time because of the seasonality of the population of *An*. *stephensi*. Larvae and pupae of *An. stephensi* and *Aedes aegypti* were visually detected but not recorded. PCR endpoint assay was performed, and successful PCR products were obtained for 130 out of 133 *Anopheles* specimens. With the PCR endpoint assay, 119 specimens were identified as *An. stephensi* and 11 specimens were identified as non*-An. stephensi*. Sequencing of portions of the ITS2 and *cox*1 loci was also completed and successful sequencing was completed for 118 *Anopheles* specimens. BLAST analysis of *Anopheles* sequences confirmed the positive detection of *An. stephensi* at all ten sites. Sequence-based species identification was mostly consistent with ITS2 endpoint assay results, except for a single specimen that was morphologically identified as *An. gambiae* (*s.l*.), identified as non-*An. stephensi* with the endpoint assay, but sequencing detected *An. stephensi*. BLAST analysis of ITS2 sequences further identified all the non-*An. stephensi* specimens as *An. arabiensis*.

**Table 3:** Distribution of *An. stephensi* based on larval breeding habitats

Region	Site	Larval breeding habitat									
		Construction water storage reservoirs (*n*)	Discarded tires (*n*)	Steel drums (*n*)	Water tanks (*n*)	Temporary water storage reservoirs (*n*)	Birkas (*n*)	Abandoned car wash (*n*)	Discarded vehicle part (*n*)	Broken bucket (*n*)	Total (*n*)
Somali	Jigjiga	nf	0	0	0	15	3	nf	nf	nf	18
	Erer	nf	0	nf	5	100	nf	24	nf	nf	129
	Kebridehar	nf	0	0	0		700	nf	nf	nf	700
	Godey	nf	0	0	0	116	224	nf	nf	nf	340
	Degehabur	nf	0	0	0	34	132	nf	nf	nf	166
Afar	Semera	225	5	0	17		13	nf	nf	nf	260
	Gewane	12	16	39	11		nf	nf	5	19	102
	Awash Sebat Kilo	26	nf	nf	0		nf	nf	nf	nf	26
Dire Dawa	Dire Dawa	100	0	24	53	100	nf	nf	nf	nf	277
Amhara	Bati	11	7	2	5	110	nf	0	nf	nf	135

*Abbreviations*: nf, not found during the survey; n, number of *An. stephensi*

We compared the morphological identification to the ITS2 PCR endpoint assay results. Of the 130 *Anopheles*, 124 were classified as *An. stephensi* and six as *An. gambiae* (*s.l*.) based on morphology. Five of the 124 (4.0%) morphologically identified *An. stephensi* were not confirmed to be *An. stephensi* with the PCR endpoint-assay. All morphologically identified *An. gambiae* (*s.l*.) (sequence-confirmed *An. arabiensis*) that were successfully amplified were also identified as non-*An. stephensi* with the ITS2 PCR endpoint assay.

All 82 wild-caught adult *An. stephensi* were screened for *P. falciparum or P. vivax* infection. No *Plasmodium* DNA was detected in any of the specimens.

## Discussion

This survey confirms that *An. stephensi* is distributed broadly in eastern Ethiopia. These data, taken with previous reports of *An. stephensi* in Kebridehar in 2016 [9], confirm that *An. stephensi* is established in this region. To our knowledge, this is the first evidence for the presence of adult *An. stephensi* in multiple regions in Ethiopia, where it might transmit malaria. The widespread presence of *An. stephensi* in Ethiopia along with Djibouti suggests that neighboring countries, such as Sudan, South Sudan, Eritrea, Somalia and Kenya, should also enhance surveillance. Given that *An. stephensi* in Djibouti was found to carry both *P. falciparum* and *P. vivax* [7, 8], there is potential for these parasites to be observed in Ethiopia; thus, malaria control strategies should now consider the potential for the established *An. stephensi* to transmit malaria.

The presence of *An. stephensi* was confirmed using both morphological and molecular methods. While morphology was mostly consistent with molecular approach (119/124 correctly identified *An. stephensi*), there were a few instances of incorrect identification based on morphology highlighting the risk for the misidentification of specimens. As more vector surveillance programmes in Africa incorporate *An. stephensi* into their morphological keys, molecular data can be helpful with the evaluation of the successful training in *An. stephensi* morphological identification. This is particularly important at this beginning phase of *An. stephensi* surveillance as field technicians will be adapting to detecting *An. stephensi*. We incorporated two molecular approaches, one of which, the end-point assay using primers designed by Djadid et al. [13] is more feasible in resource-limited settings. We found that this assay was mostly consistent with the sequence data and has to potential be integrated into current PCR based assays that focus on the detection of members of the *An. gambiae* complex, the most common malaria vectors in Africa.

While we have confirmed the broad distribution of *An. stephensi* in eastern Ethiopia, the distribution in the western part of the country is yet to be determined. West Ethiopia has had more consistent surveillance of malaria vectors than the eastern, due to the burden of the disease there; however, previously used trapping methods may limit the ability to detect *An. stephensi*. The current trapping techniques that rely heavily on CDC light traps may limit the ability to detect *An. stephensi*, given the low number of *An. stephensi* caught with CDC light traps at most sites in this study. Additional studies on the breeding, feeding, and resting behavior of *An. stephensi* can provide crucial information that can be applied to enhance future surveillance efforts in west and eastern Ethiopia.

Several additional areas of query need to be pursued further to better inform vector control efforts. No study has been published to confirm that the Ethiopian *An. stephensi* can or does transmit *Plasmodium*. Both field confirmation of infected *An. stephensi* and laboratory infections are helpful approaches evaluating this information. In the present study, the 82 wild-caught *An. stephensi* were screened for both *P. falciparum* and *P. vivax* using PCR, and *Plasmodium* was not detected. This is not unexpected as the regions included in this study report low malaria transmission, so a much larger sample size would be required to detect *Plasmodium* infection in *An. stephensi*. Future surveillance will continue to screen for *Plasmodium* using both a PCR-based and circumsporozoite protein enzyme-linked immunosorbent assay (ELISA).

The surveillance of *An. stephensi* to this point has been conducted in short time spans, with limited ability to assess changes in *An. stephensi* population size over time. We will be repeating collections in multiple sites in eastern Ethiopia to provide crucial information about how the population is changing year to year. This information will be particularly important as new vector control interventions are rolled out to evaluate their effectiveness. Insecticide resistance has been reported in the dominant malaria vector *An. arabiensis* in Ethiopia [17], but insecticide resistance status in *An. stephensi* is unknown. Investigations into insecticide resistance, the molecular mechanisms behind resistance, and potential genetic markers that can be used for surveillance are ongoing.

One question that remains unanswered relates to the origin of *An. stephensi* in Ethiopia. Previous phylogeographic analysis revealed the closest *cox*1 sequence similarity of the Ethiopia *An. stephensi* found in Kebridehar to a specimen from Pakistan [9]. Phylogeographic analysis including sequencing from recent global *An. stephensi* collections using multiple loci or whole genome sequences can help identify the exact origin of the *An. stephensi* in the Horn of Africa and how it has spread throughout the region. This information will support efforts to prevent further introduction and spread of *An. stephensi*.

An ancillary observation during the surveillance was the detection of larvae of the dengue vector *Ae. aegypti* together with *An. stephensi* larvae, suggesting that these two vectors share larval habitats. Dengue is a growing public health concern in Ethiopia, particularly in eastern Ethiopia, where major outbreaks were reported in 2013 [18, 19] and 2015 [20]. With the finding of *Ae. aegypti* larvae with *An. stephensi*, we can consider integrated vector control to target both *An. stephensi* and *Ae. aegypti.* This would be a cost-effective approach to reducing both malaria and dengue virus transmission. Future surveillance in eastern Ethiopia will work towards determining relative abundance of *Ae. aegypti* larvae at *An. stephensi* breeding sites.

## Conclusions

We confirmed that *An. stephensi* is widely distributed and established in eastern Ethiopia. Studies are ongoing to evaluate the distribution in the rest of the country and the potential risk for *An. stephensi* to change the malaria transmission landscape in the country and the rest of the African continent. Cross country cooperation and collaborations are needed to effectively address this potential global health concern.

## Data Availability

Data supporting the conclusions of this article are included within the article. All data generated or analysed during this study are available from the corresponding author upon request.

## Abbreviations

BLAST: Basic Local Alignment Search Tool
CDC: Centers for Disease Control and Prevention
*cox*1: cytochrome *c* oxidase subunit 1
DNA: deoxyribonucleic acid
ELISA: enzyme-linked immunosorbent assay
HSP: high-scoring segment pairs
ITS2: internal transcribed spacer 2
NCBI: National Center for Biotechnology Information
PCR: polymerase chain reaction
PMI: US President’s Malaria Initiative
PSC: pyrethrum spray catch
RNA: ribonucleic acid
*SSU*: small subunit
USAID: United States Agency of International Development
WHO: World Health Organization
